# Serum substance P levels are associated with severity and mortality in patients with severe traumatic brain injury

**DOI:** 10.1186/s13054-015-0911-z

**Published:** 2015-04-27

**Authors:** Leonardo Lorente, María M Martín, Teresa Almeida, Mariano Hernández, Luis Ramos, Mónica Argueso, Juan J Cáceres, Jordi Solé-Violán, Alejandro Jiménez

**Affiliations:** Intensive Care Unit, Hospital Universitario de Canarias, Ofra, s/n La Laguna, 38320 Tenerife, Spain; Intensive Care Unit, Hospital Universitario Nuestra Señora Candelaria, Carretera del Rosario s/n, 38010 Santa Cruz Tenerife, Spain; Unidad de Genética, Instituto de Enfermedades Tropicales y Salud Pública de Canarias, Universidad de La Laguna, Avenida Astrofísico Francisco Sánchez s/n, Campus de Anchieta, La Laguna, 38071 Tenerife Spain; Intensive Care Unit, Hospital General La Palma, Buenavista de Arriba s/n, Breña Alta, 38713 La Palma, Spain; Intensive Care Unit, Hospital Clínico Universitario de Valencia, Avenida Blasco Ibáñez n° 17-19, 46004 Valencia, Spain; Intensive Care Unit, Hospital Insular, Plaza Dr Pasteur s/n, 35016 Las Palmas de Gran Canaria, Spain; Intensive Care Unit, Hospital Universitario Dr Negrín, Centro de Investigación Biomédica en Red Enfermedades Respiratorias (CIBERES), Barranco de la Ballena s/n, 35010 Las Palmas de Gran Canaria, Spain; Research Unit, Hospital Universitario de Canarias, Ofra, s/n, La Laguna, 38320 Santa Cruz de Tenerife, Spain

## Abstract

**Introduction:**

Substance P (SP) is a member of the tachykinin family of neuropeptides, which are widely distributed throughout the central nervous system (CNS) and actively involved in inflammatory processes. SP is released early following acute injury to the CNS, promoting a neurogenic inflammatory response characterized by an increase in the permeability of the blood–brain barrier and the development of vasogenic edema. High levels of SP could lead to an exacerbated inflammatory response that could be fatal for patients with traumatic brain injury (TBI). Thus, the main goal of the present study was to determine whether serum SP levels are associated with injury severity and mortality in patients with severe TBI.

**Methods:**

This multicenter, observational, prospective study was carried out in six Spanish intensive care units and included patients with Glasgow Coma Scale (GCS) scores ≤8. Patients with an Injury Severity Score ≥10 in non-cranial aspects were excluded. Blood samples were collected on day 1 of TBI to measure serum SP levels. The endpoint was 30-day mortality.

**Results:**

We found higher serum SP levels (*P* =0.002) in non-surviving patients (n =27) than in surviving patients (n =73). The area under the curve for serum SP levels with regard to predicting 30-day mortality was 0.70 (95% confidence interval (CI), 0.60 to 0.79; *P* <0.001). Survival analysis showed that patients with serum SP levels >299 pg/ml had higher 30-day mortality than patients with lower levels (hazard ratio =3.7; 95% CI, 1.75 to 7.94; *P* <0.001). Multiple binomial logistic regression analysis showed that serum SP levels >299 pg/ml were associated with 30-day mortality when we controlled for APACHE II score and Marshall computed tomography lesion classification (odds ratio (OR) =5.97; 95% CI, 1.432 to 24.851; *P* =0.01) and for GCS score and age (OR =5.71; 95% CI, 1.461 to 22.280; *P* =0.01). We found a negative association between serum SP levels and GCS score (Spearman’s ρ = −0.22; *P* =0.03).

**Conclusions:**

We report, for the first time to our knowledge, that serum SP levels were associated with injury severity and mortality in patients with severe TBI. These results open the possibility that SP antagonists may be useful in the treatment of patients with severe TBI.

## Introduction

Traumatic brain injury (TBI) is a leading cause of death, disability and resource consumption [1]. There are two kinds of brain injury in TBI: primary and secondary injuries. *Primary injury* refers to the initial physical forces applied to the brain at the moment of impact and leads to shearing, laceration and stretching of nerve fibers [2,3]. *Secondary injury* is a term applied to the destructive and self-propagating biological changes in cells and tissues that lead to their dysfunction or death during the hours to weeks after the initial insult [4]. One of the process that contribute to these biological changes is neurogenic inflammation, characterized by the release of substances from primary sensory nerves, leading to vasodilation, protein extravasation and tissue swelling [5,6]. Substance P (SP), calcitonin gene-related peptide and neurokinin A are neuropeptides present in the sensory C fibers that densely surround cerebral blood vessels [7,8]. Among them, SP has been generally accepted to be associated with increased microvascular permeability and edema formation [9-11]. It is widely accepted that the development of edema has adverse consequences in TBI outcome through effects on intracranial pressure [12].

Apart from neurogenic inflammation, SP is also involved in the classical inflammatory response mediated by activation of its preferred neurokinin 1 receptor (NK1R), which is widely distributed throughout the central nervous system (CNS) [13]. Brain injury increases NK1R expression in neurons and astrocytes [14], and SP, acting through this receptor, leads to astrocyte activation. Reactive astrocytes proliferate and produce several soluble proinflammatory mediators, such as cytokines, prostaglandins and thromboxane derivatives [14-17]. In addition, both SP and NK1R are expressed in microglial cells, which are involved in initiation and/or progression of immune responses within the CNS [17]. Microglia respond to traumatic injury by migrating to the site of challenge, where they assume many of the immune effector functions typically associated with macrophages [17]. Stimulation of microglia by SP initiates activation of nuclear factor κB (NF-κB), a transcriptional activator involved in expression of proinflammatory cytokines [18]. In fact, microglia produce interleukin (IL)-1 in response to SP [19]. Apart from microglia, other brain cells can be activated by SP. In this sense, it has been shown that SP interacts with NK1R receptors present on the human neuronal cell line NT2N, inducing the expression of the potent chemokine macrophage inflammatory protein 1 [20]. In addition, SP can activate the transcription factors NF-κB and p38 mitogen-activated protein kinase in astrocyte cell lines, leading to the production of the proinflammatory cytokines IL-1, IL-6 and IL-8 [21-23]. Finally, SP can promote leukocyte chemotaxis through NK1R expressed in many inflammatory and immune cells, leading to the extravasation, migration and subsequent accumulation of leukocytes at sites of injury [5,24]. Infiltrating immune cells can contribute to the production of proinflammatory signals [25-28].

All these data suggest that SP is actively involved in the inflammatory processes following brain injury. Although SP increases after damage can be beneficial in fighting host infections associated with TBI [29], it may also play an important role in exacerbating inflammatory immune responses in the CNS, which may be fatal for patients with TBI. We therefore framed our hypothesis by testing whether serum SP levels were associated with injury severity and mortality in patients with severe TBI and whether these levels would be clinically useful in predicting mortality in these patients.

## Material and methods

### Design and subjects

This was a multicenter, observational, prospective study carried out in six intensive care units in Spain between 2009 and 2012. We included patients with severe TBI, defined as Glasgow Coma Scale (GCS) [30] score ≤8 points. We excluded individuals <18 years of age and those with pregnancy, inflammatory diseases (such as asthma, sarcoidosis, chronic obstructive pulmonary disease, inflammatory bowel disease and rheumatoid arthritis), malignant diseases or Injury Severity Score (ISS) [31] ≥10 points in non-cranial aspects. A total of 100 patients were included in the study, and they were treated according to the Brain Trauma Foundation guidelines [1].

The study was approved by the institutional review boards of the six participating hospitals: Hospital Universitario de Canarias (La Laguna, Santa Cruz de Tenerife, Spain), Hospital Universitario Nuestra Señora de Candelaria (Santa Cruz de Tenerife, Spain), Hospital General de La Palma (La Palma, Spain), Hospital Clínico Universitario de Valencia (Valencia, Spain), Hospital Insular (Las Palmas de Gran Canaria, Spain) and Hospital Universitario Dr Negrín (Las Palmas de Gran Canaria, Spain). Written informed consent was obtained from the patients or their legal guardians.

### Variables recorded

Blood samples were analyzed for glycemia, sodium, lactic acid, creatinine, bilirubin, hemoglobin, platelets, fibrinogen, international normalized ratio, activated partial thromboplastin time (aPTT) and leukocytes. Brain lesions were classified according to the Marshall computed tomography (CT) criteria [32]. To estimate the clinical severity, we calculated GCS, ISS and Acute Physiology and Chronic Health Evaluation II (APACHE II) scores [33].

The Marshall CT lesion classification scheme [32] is as follows:

- Class I or diffuse injury I: no visible pathology
- Class II or diffuse injury II: presence of cisterns with midline shift 0 to 5 mm and no high- or mixed-density lesion >25 ml
- Class III or diffuse injury III (swelling): cisterns compressed or absent with midline shift 0 to 5 mm and no high- or mixed-density lesion >25 ml
- Class IV or diffuse injury IV (shift): midline shift >5 mm and no high- or mixed-density lesion >25 ml
- Class V or evacuated mass lesion: any lesion evacuated
- Class VI or non-evacuated mass lesion: high- or mixed-density lesion >25 ml not surgically evacuated

### Endpoint

The endpoint of the study was 30-day mortality.

### Substance P assay

A total of 5 ml of venous blood samples were collected on day 1 of TBI (within the first 4 hours after TBI) through a central venous catheter. The blood was added to serum separator tubes, allowed to clot at room temperature for 30 minutes and then centrifuged at 1,000 × *g* for 15 minutes. Serum was removed and frozen at −80°C until SP measurement.

SP assay was performed in the Genetics Unit of the Instituto de Enfermedades Tropicales y Salud Pública de Canarias of the University of La Laguna (Tenerife, Spain). Serum SP levels were assayed by specific enzyme-linked immunosorbent assay according to the manufacturer’s instructions (R&D Systems, Abingdon, UK). All samples were assayed in duplicate at twofold dilutions in assay buffer. Absorbance at 450 nm was measured using the EnSpire multimode plate reader (PerkinElmer, Waltham, MA, USA). The serum concentration of SP was expressed in picograms per milliliter. The detection limit of this assay was 25 pg/ml, and the intra- and interassay coefficients of variation were 9% and 15%, respectively. Samples were all processed at the same time, at the end of the recruitment process, by the same laboratory technician using the same equipment and blinded to all clinical data.

### Statistical methods

Continuous variables are reported as medians and interquartile ranges, and comparisons between groups were carried out using the Wilcoxon-Mann-Whitney test. We used the Kolmogorov-Smirnov test to compare the frequencies distribution of our continuous variables with the theoretical normal distribution. Because lack of normality was observed in some variables included in the analysis, we used non-parametric tests to compare groups. Categorical variables are reported as frequencies and percentages, and comparisons between groups were carried out with the χ^2^ test.

Receiver operating characteristic curve analysis was carried out to determine the area under the curve (AUC). Kaplan-Meier analysis of 30-day survival was carried out using serum SP levels higher or lower than 299 pg/ml as the independent variable and survival at 30 days as the dependent variable. Comparisons were performed using the log-rank test. We used the Youden index (J) to select the cutoff point of serum SP level for the prediction of mortality at 30 days.

Multiple binomial logistic regression analyses were carried out to determine the association between serum SP levels and mortality at 30 days. In the first model, we included CT findings with high risk of death (classes III, IV and VI), APACHE II score and serum SP levels >299 pg/ml. In the second model, we included age, GCS score and serum SP levels >299 pg/ml. Odds ratios (ORs) and 95% confidence intervals (CIs) were calculated as measures of the clinical impact of the predictor variables.

A *P*-value <0.05 was considered statistically significant. Statistical analyses were performed with IBM SPSS 17.0 (IBM, Armonk, NY, USA), NCSS 2000 (Kaysville, UT, USA) and LogXact 4.1 (Cytel, Cambridge, MA, USA) software.

## Results

Comparisons of demographic and clinical severity data between surviving and non-surviving patients with TBI are shown in Table 1. We found a higher ratio of female patients among non-survivors than among survivors (11 (40.7%) of 27 vs 12 (16.4%) of 73, respectively; *P* =0.02). There were statistically significant differences in CT findings between non-surviving and surviving patients (*P* =0.002). We found a higher rate of CT findings with high risk of death (classes III, IV and VI) in non-surviving than in surviving patients (19 (70.4%) of 27 vs 26 (35.6%) of 73, respectively; *P* =0.003). Non-surviving patients with TBI had lower GCS scores (*P* <0.001), were older (*P* <0.001) and had higher APACHE II scores (*P* <0.001) than survivors. In addition, non-surviving patients had higher serum SP levels than survivors (*P* =0.002).

**Table 1:** **Comparison of demographic and clinical severity data between traumatic brain injury survivors and non-survivors** ^**a**^

	**Non-survivors (n =27)**	**Survivors (n =73)**	***P*** **-value**
Glasgow Coma Scale score	3 (3 to 6)	7 (6 to 8)	<0.001
APACHE II score	26 (25 to 32)	19 (17 to 23)	<0.001
Age (yr)	66 (45 to 76)	47 (32 to 67)	<0.001
Substance P (pg/ml)	420 (310 to 815)	250 (99 to 496)	0.002
CT findings			0.002
Class I	0	0	
Class II	3 (11.1)	21 (28.8)	
Class III	5 (18.5)	13 (17.8)	
Class IV	6 (22.2)	10 (13.7)	
Class V	5 (18.5)	26 (35.6)	
Class VI	8 (29.6)	3 (4.1)	
Female sex	11 (40.7)	12 (16.4)	0.02
Temperature (°C)	36.0 (35.0 to 37.0)	37.0 (35.6 to 37.3)	0.12
Sodium (mEq/L)	141 (135 to 149)	139 (138 to 142)	0.19
Platelets (*10^3^/mm^3^)	215 (139 to 264)	182 (143 to 252)	0.48
PaO_2_ (mmHg)	141 (104 to 186)	151 (116 to 217)	0.34
PaO_2_/FiO_2_ ratio	190 (154 to 316)	336 (242 to 407)	0.11
Leukocytes (*10^3^/mm^3^)	18.3 (10.7 to 23.9)	14.7 (10.2 to 19.3)	0.46
Lactic acid (mmol/L)	1.90 (1.15 to 4.55)	1.70 (1.23 to 2.50)	0.16
Intracranial pressure (mmHg)	20 (12 to 30)	15 (14 to 20)	0.27
International normalized ratio	1.22 (1.01 to 1.67)	1.03 (0.92 to 1.15)	0.15
Injury Severity Score	25 (25 to 27)	25 (25 to 32)	0.24
Hemoglobin (g/dl)	11.1 (9.4 to 12.3)	11.4 (10.4 to 13.0)	0.87
Glycemia (g/dl)	161 (142 to 189)	139 (120 to 163)	0.08
Fibrinogen (mg/dl)	376 (246 to 560)	350 (282 to 444)	0.32
Creatinine (mg/dl)	0.95 (0.70 to 1.10)	0.80 (0.70 to 0.90)	0.44
Cerebral perfusion pressure (mmHg)	60 (54 to 69)	68 (57 to 70)	0.46
Bilirubin (mg/dl)	0.75 (0.53 to 1.05)	0.50 (0.40 to 0.87)	0.045
aPTT (seconds)	26 (25 to 31)	28 (25 to 32)	0.86

^a^APACHE II, Acute Physiology and Chronic Health Evaluation II; aPTT, Activated partial thromboplastin time; CT, Computed tomography; PaO_2_, Partial pressure of arterial oxygen; PaO_2_/FiO_2_ ratio, Ratio of partial pressure of arterial oxygen to fraction of inspired oxygen. Data shown are number (percentage) or median (25th to 75th percentiles).

**Figure 1:**
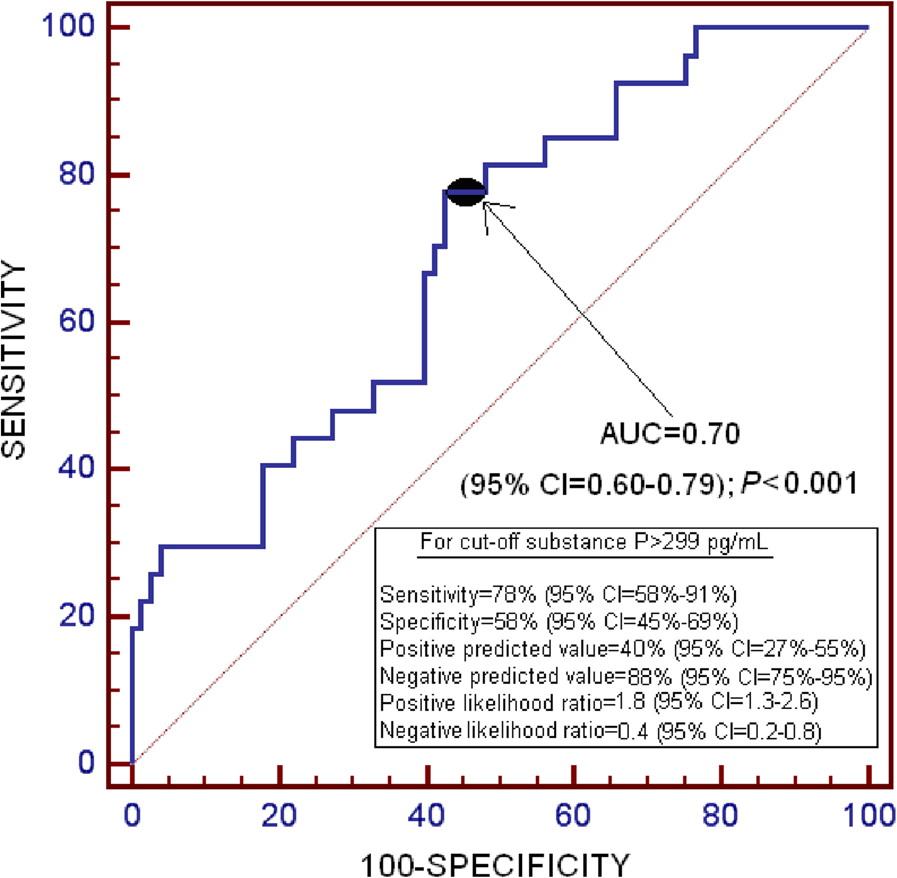
Receiver operating characteristic curve analysis of serum substance P levels to predict 30-day mortality. AUC, Area under the curve; CI Confidence interval.

The AUC for serum SP levels as a predictor of 30-day mortality was 0.70 (95% CI, 0.60 to 0.79; *P* <0.001) (Figure 1).

**Figure 2:**
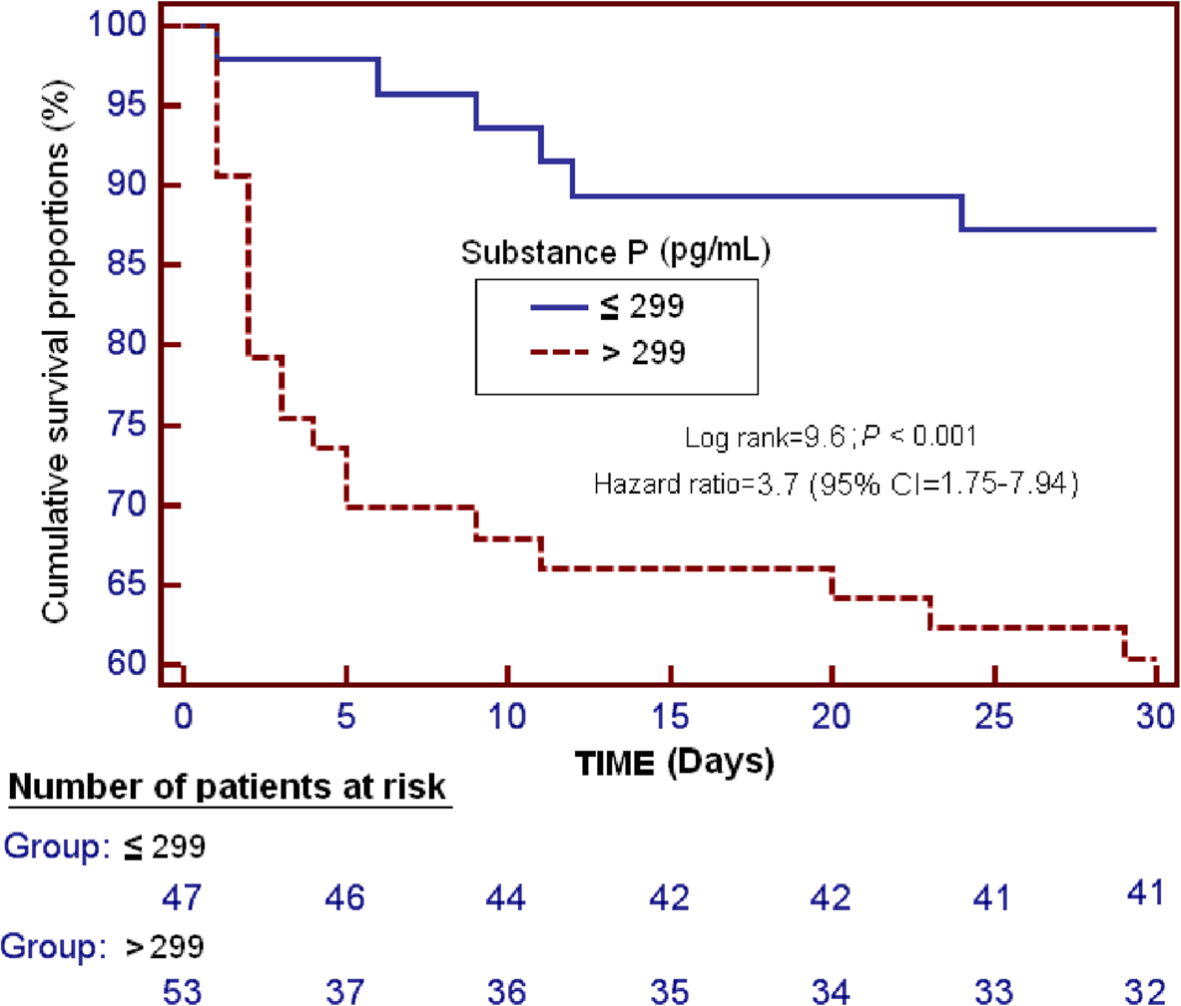
Survival curves at 30 days using serum substance P levels higher or lower than 299 pg/ml as the cutoff. CI, Confidence interval.

Survival analysis showed that patients with serum SP levels >299 pg/ml had higher 30-day mortality than patients with lower levels (hazard ratio =3.7; 95% CI, 1.75 to 7.94; *P* <0.001) (Figure 2).

Multiple binomial logistic regression analysis showed that serum SP levels >299 pg/ml were associated with 30-day mortality when we controlled for APACHE II score and CT classification (OR =5.97; 95% CI, 1.432 to 24.851; *P* =0.01) and for GCS score and age (OR =5.71; 95% CI, 1.461 to 22.280; *P* =0.01) (Table 2).

**Table 2:** **Multiple binomial logistic regression analysis to predict 30-day mortality** ^**a**^

		**Odds ratio**	**95% confidence interval**	***P*** **-value**
First model				
	APACHE II score	1.39	1.188 to 1.622	<0.001
	CT with high risk of death (classes III, IV and VI)	4.61	1.067 to 19.967	0.04
	Serum substance P levels >299 pg/ml	6.64	1.507 to 29.238	0.01
	Sex female	2.66	0.591 to 11.966	0.20
Second model				
	Age	1.08	1.029 to 1.122	<0.001
	GCS score	0.56	0.396 to 0.776	<0.001
	Serum substance P levels >299 pg/ml	7.28	1.650 to 32.086	0.01
	Female sex	3.92	0.765 to 20.118	0.10

^a^APACHE II, Acute Physiology and Chronic Health Evaluation; CT, Computed tomography; GCS, Glasgow Coma Scale.

We found no significant differences in serum SP levels between female and male patients (222 (135 to 493) vs 319 (157 to 547) pg/ml, respectively; *P* =0.35). Similarly, no association was observed between serum SP levels and intracranial pressure (Spearman’s ρ = −0.10; *P* =0.26). On the contrary, we found a negative association between serum SP levels and GCS (Spearman’s ρ = −0.22; *P* =0.03).

## Discussion

To our knowledge, this is the first study report of serum SP levels in patients with severe TBI. Our data support an important role of SP in TBI on the basis of our findings: (1) Non-surviving TBI patients showed significantly higher serum SP levels than survivors; (2) serum SP levels were significantly associated with early mortality, suggesting its potential as a biomarker; and (3) serum SP levels were significantly associated with TBI severity. Taken together, all these findings suggest that serum SP levels may be of great pathophysiological significance in patients with TBI. We also found that other variables, such as age, GCS score and CT findings, were associated with 30-day mortality, which is in agreement with previous studies [34-38].

SP stimulates mitogenesis in different cell types, including adult neural progenitor cells [39]; increases axonal growth from the dorsal horn [40]; and promotes neurite outgrowth [41]. In spite of this beneficial action on neurogenesis, several studies carried out in animal models support a deleterious role of SP in TBI. In rats, SP release after severe diffuse TBI increased vascular permeability and edema formation, both of which were attenuated after administration of a NK1R antagonist [10,11]. SP can promote proliferation [42], chemotaxis [43] and activation of microglia [18,25], and the subsequent release of proinflammatory cytokines by activated cells may impair neurogenesis and motor outcome [44-46]. In fact, treatment of rats with a NK1R antagonist after TBI improved functional outcome [42]. In addition, proinflammatory mediators can be released by other brain cells expressing NK1R in response to SP, such as astrocytes and neuronal cells, contributing to an increase in the inflammatory response [20-23]. Finally, capsaicin, a substance that induces SP depletion from sensory nerves, administered to rats before TBI attenuated blood brain–barrier opening, edema formation and the development of both motor and cognitive deficits [6]. These results obtained in small animal models have been reproduced using an ovine model of TBI [47]. In this model, administration of a NK1R antagonist decreased edema formation and caused a profound reduction in posttraumatic intracranial pressure. In humans, a recent study carried out with archival postmortem material found increased SP immunoreactivity in different brain areas of patients who experienced TBI compared with patients with no neuropathological abnormality [7].

Taking into account all these data, we proposed that brain injury in humans induces SP release from sensory neurons, which trigger a cascade of events that results in activation of NK1R in cerebral blood vessels, leading to increased vascular permeability and edema formation. In addition, activation of neurons, astrocytes and microglia by SP leads to inhibition of neurogenesis and chemotaxis of inflammatory and immune cells to the site of injury. Inflammatory cells may use SP as a paracrine or autocrine signaling mechanism to propagate inflammation beyond the limited topographic spread of the sensory neurons [48]. The concentration of neuroimmune SP increases in the brain, leaking into the bloodstream, where it can be quantitated in serum or plasma samples. This measurement would allow identification of those patients with an exacerbated inflammatory response. This is particularly interesting because NK1R antagonists have been approved for commercial use to treat different pathologies [49-51], and they could be an attractive therapeutic option for these patients [52].

The observed association of SP with higher mortality may be due to malignant and inflammatory conditions that are known to increase SP levels [53-55]. However, patients with diseases such as asthma, sarcoidosis, chronic obstructive pulmonary disease, inflammatory bowel disease, rheumatoid arthritis or malignant diseases were not included in the present study. Even so, we cannot discount the possibility that other conditions may influence SP levels. Elevated serum SP levels could reflect greater injury severity and hence higher mortality. However, multiple logistic regression analysis showed that serum SP levels were associated with mortality after we controlled for GCS and APACHE II scores and age.

Previous studies have shown that serum SP levels vary considerably over time after TBI [10]. Thus, as soon as 30 minutes after injury, a significant increase in serum SP levels was observed in mice, but these levels decreased by 5 hours, presumably due to rapid proteolysis by non-specific serum proteases [10]. In our study, all samples were collected within 4 hours after TBI, although the exact interval between the injury, hospital admission and blood collection were not recorded. In addition, we did not analyze serum SP levels during follow-up. Therefore, information regarding sample collection period and patient monitoring would be desirable in future studies to determine the role of SP in TBI.

Although the mortality rate in our series (27%) is similar to that reported in other studies [56,57], it should be noted that the mortality rate may be different in series with a different case mix (that is, with regard to GCS, APACHE II, age and CT findings). Moreover, owing to the low number of events (deaths), we included only three variables in the multiple logistic regression analysis to avoid a final model of order slightly higher than required due to an overfitting effect [58]. In addition, in the multiple regression analysis, we included those variables that showed statistically significant differences in the bivariate analysis when survivors and non-survivors were compared. We also used two models to avoid the effects of collinearity. Thus, we constructed two multiple binomial logistic regression models with only three predictor variables in each model, including serum SP levels in both models. One model controlled for CT findings and APACHE II score, and the other controlled for age and GCS score. We found that serum SP levels were associated with mortality in both models, which strongly supports the role of SP in TBI. However, larger series of patients and the inclusion of more variables in a single multiple logistic regression analysis are needed to confirm our findings.

## Conclusions

The most relevant findings of our study, which is the first to include data on serum SP levels in patients with severe TBI to our knowledge, are that non-surviving TBI patients showed higher serum SP levels than survivors, that serum SP levels were associated with severity and mortality, and that serum SP levels could be used as a biomarker to predict mortality in patients with severe TBI. These results open the possibility that NK1R antagonists may be useful for the treatment of severe TBI.

## Key messages

- Serum substance P levels are associated with TBI severity.
- Non-surviving TBI patients showed higher serum substance P levels than survivors.
- Serum substance P levels are associated with TBI mortality.

## Abbreviations

APACHE: Acute Physiology and Chronic Health Evaluation
aPTT: Activated partial thromboplastin time
AUC: Area under the curve
CI: Confidence interval
CNS: Central nervous system
CT: Computed tomography
FiO_2_: Fraction of inspired oxygen
GCS: Glasgow Coma Scale
IL: Interleukin
ISS: Injury Severity Score
NF-κB: Nuclear factor κB
NK1R: Neurokinin 1 receptor
OR: Odds ratio
PaO_2_: Partial pressure of arterial oxygen
SP: Substance P
TBI: Traumatic brain injury
